# Antitumour activity of novel taxanes that act at the same time as cytotoxic agents and P-glycoprotein inhibitors

**DOI:** 10.1054/bjoc.2000.1500

**Published:** 2000-12

**Authors:** C Ferlini, M Distefano, F Pignatelli, S Lin, A Riva, E Bombardelli, S Mancuso, I Ojima, G Scambia

**Affiliations:** Laboratory of Antineoplastic Pharmacology, Department of Obstetrics and Gynaecology, Università Cattolica Sacro Cuore, Rome, Italy; Indena, Spa, Milan, Italy; Department of Chemistry, State University of New York at Stony Brook, Stony Brook, NY, USA

**Keywords:** P-glycoprotein, cell cycle block, apoptosis, paclitaxel, taxanes

## Abstract

Taxanes antitumour agents such as paclitaxel and docetaxel represent a successful family of chemotherapeutic drugs. Unfortunately, acquired and innate resistance represents a clinical problem for these drugs. We investigated, on a panel of 7 human cancer cell lines, the growth inhibition effect of 3 newly developed taxanes (SB-T-1213, SB-T-1250 and SB-T-101187) with modification at the C10 and C3′ positions of the taxane framework. These positions have been previously characterized as critical to make taxanes highly active against cells overexpressing the efflux pump P-glycoprotein (P-gp). Paclitaxel and docetaxel were used as reference compounds. Results unambiguously indicate the exceptional activity of the novel taxanes toward P-gp positive cells (up to >400 fold higher potency than that of paclitaxel). SB-T-1213 and SB-T-1250 are also substantially more active than the reference compounds against P-gp negative cells. To better understand the mechanisms underlying the enhanced activity of the newly developed taxanes, we performed cell cycle and apoptosis analysis. This study demonstrates that the striking growth inhibition effect exhibited by the novel taxanes is ascribed to their increased ability in inducing apoptosis and G _2_/M cell cycle block. SB-T-1213 and SB-T-1250 are also more active than reference compounds in inducing intracellular accumulation of the beta-tubulin subunits. Finally, it is revealed that these novel taxanes have ability to inhibit the function of the P-gp efflux pump on the basis of the Rhodamine 123 assay. These findings strongly suggest that SB-T-1213, SB-T-1250 and SB-T-101187 represent a new tool to overcome innate or acquired P-gp mediated taxane-resistance. © 2000 Cancer Research Campaign http://www.bjcancer.com


*British Journal of Cancer* (2000) **83**(12), 1762–1768
© 2000 Cancer Research Campaign
doi: 10.1054/ bjoc.2000.1500, available online at http://www.idealibrary.com on IDEAL®

http://www.bjcancer.com

### Antitumour activity of novel taxanes that act at the same time as cytotoxic agents and P-glycoprotein inhibitors

**C Ferlini[1], M Distefano[1], F Pignatelli[1], S Lin[2], A Riva[3], E Bombardelli[3], S Mancuso[1], I Ojima[2] and G Scambia[1]**

[1]Laboratory of Antineoplastic Pharmacology, Department of Obstetrics and Gynaecology, Università Cattolica Sacro Cuore, Rome, Italy; [2]Indena, Spa, Milan, Italy; [3]Department of Chemistry, State University of New York at Stony Brook, Stony Brook, NY, USA

**Summary** Taxanes antitumour agents such as paclitaxel and docetaxel represent a successful family of chemotherapeutic drugs. Unfortunately, acquired and innate resistance represents a clinical problem for these drugs. We investigated, on a panel of 7 human cancer cell lines, the growth inhibition effect of 3 newly developed taxanes (SB-T-1213, SB-T-1250 and SB-T-101187) with modification at the C10 and C3′ positions of the taxane framework. These positions have been previously characterized as critical to make taxanes highly active against cells overexpressing the efflux pump P-glycoprotein (P-gp). Paclitaxel and docetaxel were used as reference compounds. Results unambiguously indicate the exceptional activity of the novel taxanes toward P-gp positive cells (up to >400 fold higher potency than that of paclitaxel). SB-T-1213 and SB-T-1250 are also substantially more active than the reference compounds against P-gp negative cells. To better understand the mechanisms underlying the enhanced activity of the newly developed taxanes, we performed cell cycle and apoptosis analysis. This study demonstrates that the striking growth inhibition effect exhibited by the novel taxanes is ascribed to their increased ability in inducing apoptosis and $G_2$/M cell cycle block. SB-T-1213 and SB-T-1250 are also more active than reference compounds in inducing intracellular accumulation of the beta-tubulin subunits. Finally, it is revealed that these novel taxanes have ability to inhibit the function of the P-gp efflux pump on the basis of the Rhodamine 123 assay. These findings strongly suggest that SB-T-1213, SB-T-1250 and SB-T-101187 represent a new tool to overcome innate or acquired P-gp mediated taxane-resistance. © 2000 Cancer Research Campaign http://www.bjcancer.com

**Keywords**: P-glycoprotein; cell cycle block; apoptosis; paclitaxel; taxanes

Taxanes constitute a family of naturally occurring diterpene compounds including a potent antitumour drug, paclitaxel. Paclitaxel, originally isolated from the bark of the Pacific Yew tree (*Taxus brevifolia*), has been shown highly effective in adjuvant and neo-adjuvant therapies for patients with breast and ovarian cancers. More recently, its semisynthetic analogue, docetaxel, has been introduced to breast cancer chemotherapy, which appears to have expanded the number of diseases potentially sensitive to this class of antitumour drugs. Both paclitaxel and docetaxel bind to tubulin (Manfredi et al, 1982; Rao et al, 1992), inhibit microtubule-disassembly (Schiff et al, 1979) and impair mitosis (Long and Fairchild, 1994), thereby blocking progression through M phase of the cell cycle and facilitating apoptosis (Jordan et al, 1996). In spite of the undoubted clinical success, some tumours display innate or acquired resistance to these drugs. Three main mechanisms of drug-resistance have been reported, (i) point mutations of the tubulin gene (Bhalla et al, 1994), (ii) selection of tubulin isoforms with low binding to taxanes (Kavallaris et al, 1997; Ranganathan et al, 1998) and (iii) expression of the multidrug-resistance (MDR) phenotype mediated by the P-glycoprotein (P-gp) efflux pump encoded by the mdr1 (Horwitz et al, 1993; Chevillard et al, 1996; Mechetner et al, 1998). The mechanism (iii) contributes to explain the innate resistance to paclitaxel and docetaxel for tumours that often inherently express P-gp, such as colon and kidney cancers (Naito and Tsuruo, 1998; Yu et al, 1998; Gao et al, 1999). P-gp seems to be involved in the drug-resistance of primary or metastatic tumours of the CNS, where molecules very similar to P-gp (P-gp sister proteins) contribute to the generation of the brain–blood barrier and hamper the trafficking of the bulky hydrophobic molecules such as paclitaxel and docetaxel (Herrington et al, 1998).

In order to discover and develop new taxanes active against MDR-positive cells, a good number of taxanes derived from naturally occurring 10-deacetylbaccatin III as well as 14-hydroxy-10-deacetylbaccatin III have been designed, synthesized, and assayed for their activities against P-gp positive cell lines (Ojima et al, 1996; Distefano et al, 1997; Ojima et al, 1998). Structure–activity relationship study demonstrated that certain modifications at the C10/C3′ positions of the taxane skeleton lead to the compounds possessing high activity against MDR-positive cell lines (Ojima et al, 1996, 1997a, 1997b). In the present study, we selected three of these novel and highly active taxanes to investigate their pattern of antitumour activity in comparison with paclitaxel and docetaxel. This study clearly shows that newly developed taxanes exhibit a spectrum of antitumour properties not fully overlapping with and significantly better than those of paclitaxel and docetaxel, justifying for these compounds to be defined as the second-generation taxanes.

*Received 15 June 2000*
*Revised 7 August 2000*
*Accepted 13 August 2000*

*Correspondence to:* G Scambia

### MATERIALS AND METHODS

### Drugs

Docetaxel and paclitaxel were provided by Indena (Milan, Italy), dissolved in DMSO (stock solution 10 mM) and used within 7 days. Taxanes SB-T-101187, SB-T-1213 and SB-T-1250 were synthesized using the procedures previously reported (Ojima et al, 1997a, 1997b, 1998) and used as DMSO solution in the same manner as mentioned above. Chemical structures of all the drugs used in this study are shown in Scheme 1. Control cells were treated with the same amount of vehicle alone. The final DMSO concentration never exceeded 0.1% (v/v), in either control or treated samples.

### Cell culture

A panel of seven human cancer cell lines was used. MCF-7wt, PC-3, DU145, NCIH23 and WiDr cells were purchased from the American Type Culture Collection (Rockville, MD); the multidrug resistant (MDR) MCF-7 ADRr line, selected for adriamycin resistance was kindly provided by Dr Kenneth H Cowan (National Cancer Institute, NIH, Bethesda, MD). A2780 cells were purchased from the European Collection of Cell Cultures (ECACC, Salisbury, UK). Culture media were chosen according to the suggestions of ATCC and ECACC. MCF-7 ADRr cells were cultured in RPMI 1640 supplemented with 10 μM of ADR. Cells, propagated as monolayer culture in 75 $cm^2$ tissue-culture flasks, were trypsinized once or twice (depending on the cell type) weekly and plated at a density of $8 \times 10^4$ cells $ml^{-1}$. All cultures were incubated at 37°C under 5% $CO_2$ 95% air in a high humidity atmosphere. MCF-7 ADRr cells exhibit the classical multidrug-resistance (MDR) phenotype with mdr-1 mRNA and P-gp overexpression (De-Vincenzo et al, 1996). Experiments on P-gp function were performed also in CEM VBLr cells (gift from M Cianfriglia, Istituto Superiore di Sanità, Rome), an additional cell line expressing the MDR phenotype. Cells were cultured in RPMI 1640 as indicated for MCF-7 ADRr cells.

### Growth experiments

Cells were plated in 6-well flat bottom plates (Falcon, Lincoln Park, NJ) at a concentration of $10^5$ cells $ml^{-1}$ in complete medium. After 24 h, the medium was replaced with media containing the test substances. Control cells were treated with vehicle alone. Cells were then washed twice and cultures maintained up to 72 h in complete medium (without removing drugs). At this time point quadruplicate counts of triplicate cultures were performed. To calculate the growth inhibition effect, three independent experiments were performed for each drug/cell line. The $IC_{50}$ values were then calculated by fitting the concentration–effect curve data obtained in the three experiments with the sigmoid-Emax model using nonlinear regression, weighted by the reciprocal of the square of the predicted effect (Motulsky and Ransnas, 1987).

### DNA analysis, tubulin expression and assessment of apoptosis

Cells were plated at a concentration of $10^5$ cells $ml^{-1}$ in the specific medium supplemented as above. After 24 h, the medium was replaced with fresh one containing the compounds to be tested or vehicle alone. After various times of culture (from 24 h to 72 h) cells were harvested and nuclei isolated and stained using a solution containing 0.1% $Na^+$ citrate, 0.1% NP40, 4 mM EDTA and 50 μg $ml^{-1}$ propidium iodide (PI) as DNA dye (Ferlini et al, 1996). Incubation of the cells with staining solution lasted a minimum of 12 h at 4°C. Flow cytometric DNA analysis was performed by acquiring a minimum of $2 \times 10^4$ nuclei with a Facscan flow cytometer (Becton Dickinson, San Jose, CA). DNA fluorescence was collected in linear mode and pulse signal processing was used to set a doublet discrimination gate. Cell cycle analysis was performed using Multicycle software package (Phoenix, San Diego, CA). In other experiments, DNA analysis was performed in whole cells in dual staining with microtubules. Briefly, cells were fixed in cold methanol for 10 min at –20°C. After one washing pellets were re-suspended in cold acetone for 20 s. After two washings, cells were labelled either with a FITC-conjugated monoclonal antibody anti-α (diluted 1:50) or anti-β (diluted 1:25)

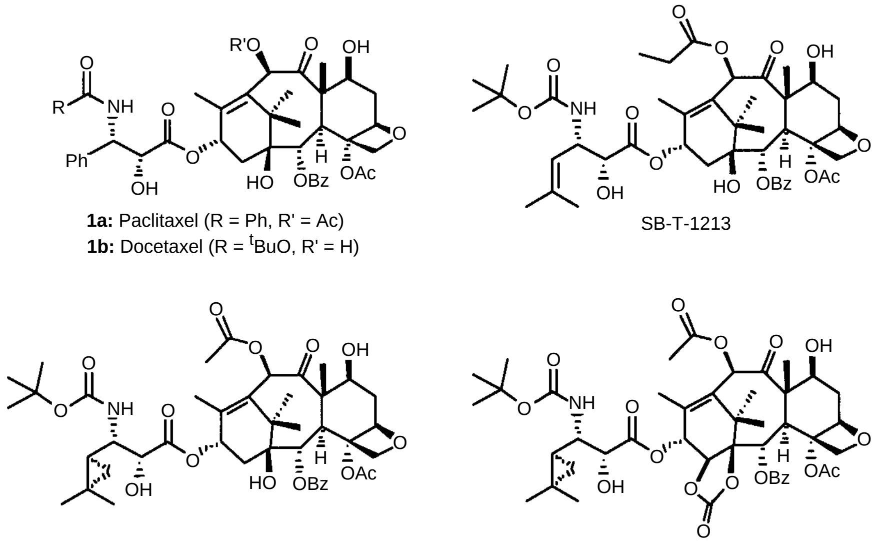

**Scheme 1**

tubulin (Sigma, St Louis, MO). After 1 h of incubation at 4˚C samples were washed twice and maintained at 37˚C for 20 min in PBS containing 50 U $ml^{-1}$ of RNAse A (Sigma). Finally, PI at a final concentration of 50 µg $ml^{-1}$ was added and incubation lasted a minimum of 12 h at 4˚C. The expression of tubulin subunits (in triplicate samples) was calculated in cells gated in $G_{0/1}$ and $G_2$/M phases of the cell cycle. The mean channel of FITC fluorescence was calculated for each condition. The mean of untreated cells represented the 100%. Data are presented as the percent of the control cells ± SD of the triplicates.

The Apodirect kit (Pharmingen, San Diego) that includes all the reagents to perform the d-UTP labelling of DNA strand breaks measured Apoptosis. Kit was used following the manufacturer's suggestions. Apoptosis was determined in two independent experiments in WiDr cells cultured for 3, 6, 12 and 24 h in the presence of taxanes (at 1 and 2.5 nM, with exception of paclitaxel that was used at 2.5 and 5 nM).

### *Assessment of P-gp function*

Rhodamine 123 (Rh123) fluorescent probe (Molecular Probes, Eugene, OR) served to measure the functionality of the P-gp efflux pump. Briefly, MCF-7 ADRr and CEM VBLr cells were loaded at 37˚C with 0.5 µM of the dye in PBS supplemented by 0.2% of BSA. After 15 min cells were transferred onto ice and washed two times to remove Rh123 from the medium. After washing, 10 µM of the potential P-gp inhibitors was added and cells were kept at 37˚C over the period of 20 to 80 min. The positive control was represented by quercetin (Sigma), a well known inhibitor of the P-gp function. An aliquot of cells was maintained on ice to prevent dye efflux (control at 4˚C), whereas the maximal efflux was established by adding the vehicle DMSO 0.1% and allowing the efflux at 37˚C. Flow cytometry served to measure Rh123 fluorescence, by acquiring 15 000 cells with a Facscan flow cytometer equipped with standard filters for green signal. Mean channel of the Rh123 fluorescence was calculated for each condition. The ratio of mean channel between control at 37˚C and control at 4˚C was considered the control dye efflux. Similarly, the mean channel of Rh123 fluorescence of treated cells was divided over the control at 4˚C. This ratio was divided over the control dye efflux to establish the potency of P-gp inhibition. Results coming from two independent experiments have been averaged and standard deviations are reported in error bars.

### RESULTS

### Growth inhibition effect

Growth inhibition effects of SB-T-1213, SB-T-1250 and SB-T-101187 on cancer cells were assessed against a panel of seven human cancer cell lines. Results are summarized in Table 1. When considering results of the taxanes currently used in clinic, docetaxel is always more active than paclitaxel (from 1.1 to 3.4 folds). SB-T-101187 shows a growth inhibition effect between docetaxel and paclitaxel, with the exception of MCF-7 ADRr and PC-3 cells. SB-T-1250 and SB-T-1213 showed a striking effect against all cell lines, exhibiting substantially higher potencies than that of paclitaxel (from 1.8 to 69-fold for SB-T-1250 and 3 to 412-fold for SB-T-1213).

### DNA analysis and apoptosis

In order to test if the gradient in antitumour activity of these novel taxanes is parallel to the blocking activity, we have performed DNA analysis in WiDr cells. After 24 hours of culture, the taxane-treatment of WiDr cells blocks the cell cycle at the $G_2$/M phase (Figure 1). The increase in the number of blocked cells appears dose dependent for all taxanes and the higher extent of cell cycle block is observed at 5 nM for all drugs. After 72 hours of culture, a large part of blocked cells exits from the M phase. This exit is followed by an increase of DNA fragmentation, suggesting that the majority of blocked cells undergo apoptosis, as previously demonstrated for paclitaxel and docetaxel in other cell lines. However, the number of cells showing hypo-diploid DNA content does not correlate with the block. The largest amount of fragmented DNA is observed at concentrations lower than 5 nM. For paclitaxel, docetaxel and SB-T-101187 the peak of DNA fragmentation occurs at 2.5 nM, while for SB-T-1213 and SB-T-1250 the peak appears at 1 nM.

To ascertain the actual amount of cells undergoing apoptosis, we chose a method to score apoptosis that allows the identification of the cell cycle phase from which the cell death process stems. The technique (tunel assay) is based on the labelling of DNA-strand breaks (occurring in cells undergoing early apoptosis) by the terminal deoxynucleotide transferase enzyme that incorporates a marked nucleotide (BrDu-FITC) in the DNA chain (Darzynkiewicz et al, 1997). The assay was performed after 3, 6, 12 and 24 hours of the WiDr cell culture in the presence of taxanes. Figure 2 shows a representative analysis of the most active agent SB-T-1213. The exposure of DNA-strand breaks starts from 3 and progressively increases up to 12 hours. After an additional 12 hours of culture, a smaller number of cells with exposed DNA-strand breaks becomes detectable. Concomitantly, a subset of cells with a DNA content higher than 4C (indicated by double arrows in Figure 2E) comes into the view, along with the increase in the number of hypo-diploid cells (indicated by arrows in Figure 2E). Interestingly, these cells were completely devoid of DNA-strand breaks in this system. By this method, we were able to score the actual amount of apoptosis (length of bars in Figure 3). The ability of the taxanes in inducing apoptosis followed the same order of potency observed for the cell cycle-block and the growth inhibition effect. Moreover, we observed that the maximal occurrence of DNA-strand breaks started from the $G_2$/M phase of the cell cycle.

**Table 1** $IC_{50}$ values (expressed in nM) against a panel of human cancer cell lines

| Drug | A2780 | MCF-7 wt | MCF-7 ADRr | WiDr | PC-3 | DU-145 | NCI-H23 |
|---|---|---|---|---|---|---|---|
| Paclitaxel | 5.4 | 5.8 | 3300 | 3.8 | 3.4 | 1.5 | 4.1 |
| Docetaxel | 4.95 | 3.6 | 970 | 2.3 | 1.8 | 0.8 | 3 |
| SB-T-101187 | 5.16 | 5.6 | 108 | 3 | 3.8 | 0.7 | 2.9 |
| SB-T-1250 | 3.03 | 2.3 | 48 | 0.5 | 0.7 | 0.6 | 1.6 |
| SB-T-1213 | 1.77 | 0.9 | 8 | 0.4 | 0.3 | 0.3 | 0.7 |

 *© 2000 Cancer Research Campaign*

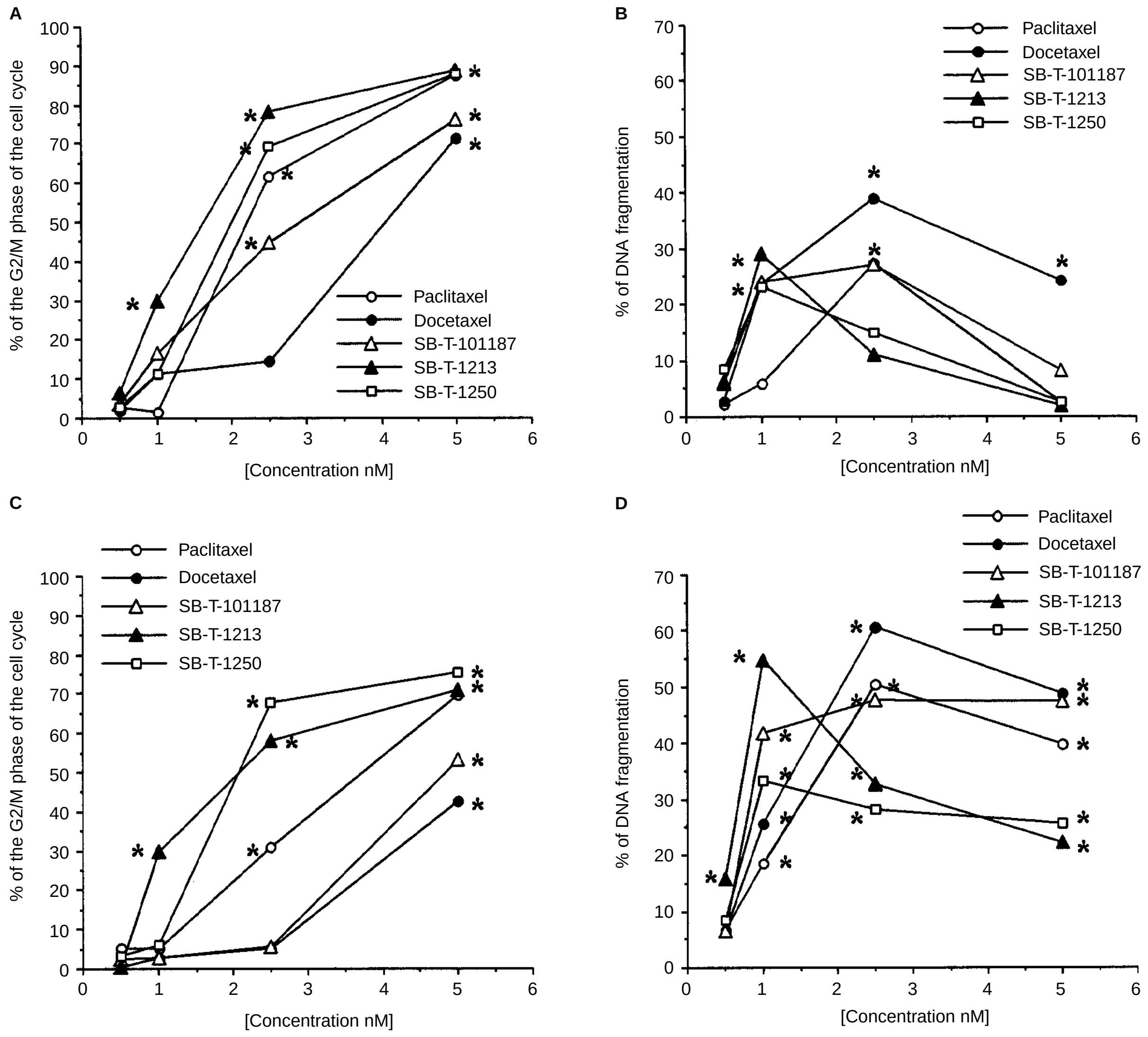

**Figure 1** Line charts showing results of DNA analysis in WiDr cells cultured for 24 (**A–B**) and 72 (**C–D**) hours in the presence of taxanes (concentration range 0.5–5 nM). In the graphs **A** and **C** the percent of cells blocked in G2/M phase of the cell cycle is shown. In the graphs **B** and **D**, the amount of events in the hypo-diploid region is depicted. The following symbols are used: open circle (paclitaxel), black circle (docetaxel), open triangle (SB-T-101187), black triangle (SB-T-1213), and open square (SB-T-1250). SD bars have been omitted to add clarity to the graphs. *$P < 0.05$ vs. the control

All these findings indicate that apoptosis is triggered, in a dose-dependent manner, in cells blocked at $G_2$/M phase of the cell cycle. However, the DNA release, resulting in the generation of hypo-diploid cells, is hampered when concentrations higher than 1 nM of SB-T-1213 or SB-T-1250 and 2.5 nM of the other taxanes are used. This phenomenon can be likely ascribed to the stiffening of the microtubule network.

### Analysis of tubulin expression

Flow cytometry was used to evaluate the expression of α- and β-tubulin isotypes in WiDr cells treated with 1 and 2.5 nM of taxanes for 24 hours. Analysis was performed in dual staining with PI as DNA dye. This method allowed us to assess tubulin expression in cells gated in the $G_{0/1}$ and $G_2$/M phase. The drug-treatment does not affect α-tubulin significantly at both concentrations, whereas results for β-tubulin isotype are summarized in Figure 4. β-tubulin is upregulated by SB-T-1213 and SB-T-1250 at 1 nM without differences between cells gated at $G_{0/1}$ and $G_2$/M. Increase in taxane concentration induces the augmentation of β-tubulin for the remaining drugs. There is no significant difference between cells gated in $G_{0/1}$ and $G_2$/M in this case as well. A tall conditions examined, the increase never exceeds 3-fold of the value of control cells, indicating the saturation in the tubulin-binding of taxanes.

### Effect of newly developed taxanes on P-gp function

The functional activity of the P-gp efflux pump was firstly investigated by incubating MCF-7 ADRr cells (loaded with the cationic probe Rh123) in the presence of 10 μM of SB-T-1213, SB-T-1250 and SB-T-101187. Three types of controls were used, (i) cells kept on ice to prevent the active efflux of Rh123 (maximal loading); (ii) cells maintained at 37°C to allow the dye efflux (maximal efflux); (iii) cells treated by 10 μM of quercetin, a well known inhibitor of the P-gp function (Shapiro and Ling, 1997). Cells treated by

© 2000 Cancer Research Campaign

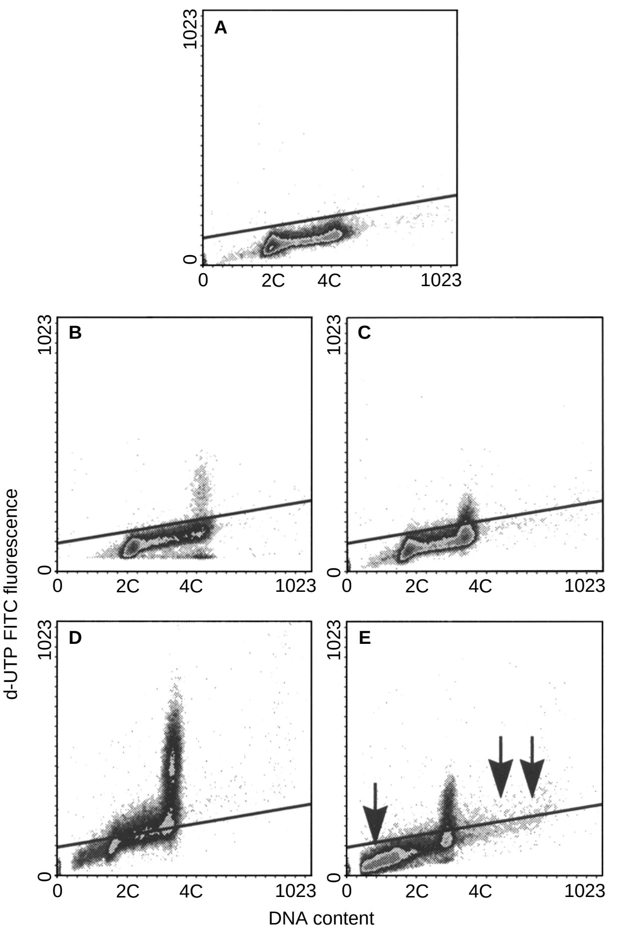

**Figure 2** Representative dot density plots of the DNA content vs. d-UTP fluorescence in WiDr cells cultured in the presence of SB-T-1213 for 3 (**B**), 6 (**C**); 12 (**D**) and 24 (**E**) hours of culture. In the panel **A**, control cells at 3 hours is shown. Cells shown above the line are considered d-UTP positive. Markers to set regions of 2C and 4C DNA are shown below the X-axis. Arrows in the panel E indicate the increase in the number of cells in the hypo-diploid region (all d-UTP negative). Double arrows indicate the presence of cells able to recycle from the 4C region of the cell cycle quercetin and taxanes were maintained at 37˚C to allow the maximal dye efflux. Results are summarized in Figure 5. SB-T-1213 and SB-T-101187 are the most potent agents among the examined drugs. Moreover, it should be noted that the kinetic of P-gp inhibition by SB-T-1213 and SB-T-101187 appears different from that of quercetin, i.e., the activity of SB-T-1213 and SB-T-101187 decreases as the time goes by whereas P-gp inhibition of quercetin is stable during the time course. SB-T-1250 also shows substantially weaker inhibitory activity of P-gp function as compared to those of SB-T-1213 and SB-T-101187. As anticipated, docetaxel is completely inactive for the modulation of P-gp function. In order to confirm these findings, we repeated the same experiments in an additional P-gp positive cell line (CEM VBLr), confirming the results in terms of P-gp inhibition (data not shown).

### DISCUSSION

The introduction of paclitaxel and docetaxel in cancer chemotherapy has significantly improved the clinical course of diseases such as ovarian and breast cancers (Verweij et al, 1994). However, some tumour histotypes such as those in colon, kidney and CNS appear to be poorly responsive to this class of drugs. Unfortunately, the same phenomena occur in the cancer patients relapsing after taxane treatment. New hopes to overcome these problems may stem from our present study. We examined the profile of the antitumour properties of 3 newly developed taxanes SB-T-1213, SB-T-1250 and SB-T-101187. It is noteworthy that SB-T-1213 and SB-T-1250 have exhibited strikingly high potency in all the biological effects examined. These two novel taxanes can block cell cycle, hamper β-tubulin depolymerization and induce apoptosis at a concentration 2.5 times lower than that required by the other taxanes, including paclitaxel and docetaxel. Moreover, we have discovered the mechanism underlying their excellent activity against P-gp positive cells. These novel taxanes act as cytotoxic agents and at the same time inhibit the function of the P-gp efflux pump. This property is absolutely unique in the field of the antitumour agents and could produce several potential advantages: (i) overcoming drug resistance mediated by the expression of the MDR phenotype; (ii) possessing strong activity in those cancers that inherently express low levels of the P-gp pump (such as colon and kidney cancers); (iii) possible synergistic combinations with other antitumour agents such as anthracyclines that are substrates for the P-gp protein. Previous attempts to modulate P-gp function using a two-drug approach (such as r-verapamil, tamoxifen) failed to produce positive clinical results, and these findings contributed to the current thinking that P-gp is not a relevant cause of drug-resistance in solid tumours (Kaye, 1998). However, it is possible that interpretation of the results from these trials is complicated by pharmacokinetic interactions between the target cytotoxic drug and the P-gp-reverting agent and the use of a single drug with intrinsic ability of P-gp inhibitor will contribute to understand which is the actual relevance of P-gp in the development of drug-resistance. It is also anticipated that these novel taxanes may well have more diverse pharmacokinetic profile as compared to those of paclitaxel and docetaxel, because P-gp belongs to a superfamily of highly-conserved proteins, named ABC, ATP Binding Cassette, that are responsible for the vast majority of trafficking of the bulky hydrophobic molecules (including intestinal absorption and crossing of the haemato-encephalic barrier) (Torok et al, 1999). On the other hand, the interaction with the P-gp function could increase toxicity towardhaematopoietic progenitors and produce adverse effects in the most immature haematopoietic cells. Indeed, haematopoietic progenitors express on their membranes P-gp in a manner to protect this precious cell source from noxious xenobiotics (Smeets et al, 1997). The protein is highly expressed at the level of the most immature $CD34^+$ cells, whereas during the course of differentiation the pump is gradually lost (Uchida et al, 1996). Thus, the assessment of toxicity in the haematopoietic compartment is mandatory prior to hypothesizing a possible clinical use. Nevertheless, the remarkable antitumour properties and the ability to inhibit P-gp function described here warrant further investigations to identify the mechanism(s) of the P-gp inhibition by these novel taxanes as well as the pharmacokinetic properties in animal models. These studies will further advance the development of a 'second generation' of taxanes, hoping to obtain drugs with an enhanced spectrum of activity and improved pharmacological properties.

 © 2000 Cancer Research Campaign

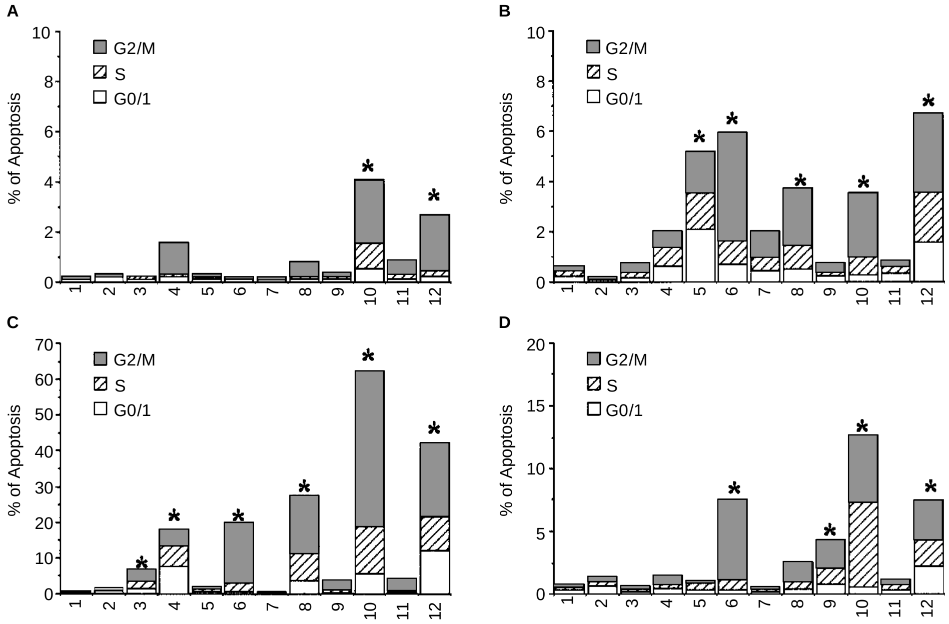

**Figure 3** Stack column bars show the results of apoptosis analysis after 3 (**A**), 6 (**B**), 12 (**C**) and 24 (**D**) hours of culture. Cells labelled with d-UTP are considered as apoptotic. These cells were gated and then the cell cycle analysis was performed. Lanes 1 (control), 2 (DMSO 0.1 %), 3 (paclitaxel 2.5 nM), 4 (paclitaxel 5 nM), 5 (docetaxel 1 nM), 6 (docetaxel 2.5 nM), 7 (SB-T-101187 1 nM), 8 (SB-T-101187 2.5 nM), 9 (SB-T-1213 1 nM), 10 (SB-T-1213 2.5 nM), 11 (SB-T-1250 1 nM), 12 (SB-T-1250 2.5 nM). Results represent the mean of two independent experiments. *$P < 0.05$ vs. the control

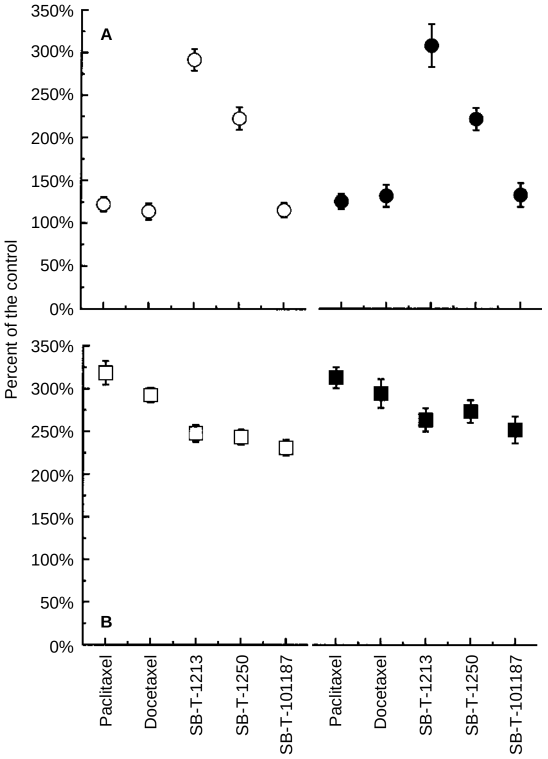

**Figure 4** Box plots show the results of tubulin analysis after 24 hours of culture in the presence of 1 nM (**A**, circle) and 2.5 nM (**B**, square) of taxanes. Samples were prepared in triplicate for each condition. Symbols and bars are mean and standard deviations of the triplicates, respectively. Results in y-axis are expressed as percentage of the control (DMSO 0.1%). Open and closed symbols are referred to as cells gated at the $G_{0/1}$ and $G_2$/M phase of the cell cycle, respectively. Interestingly, when the alteration of the beta tubulin expression occurs, the over expression also takes place at the $G_{0/1}$ phase

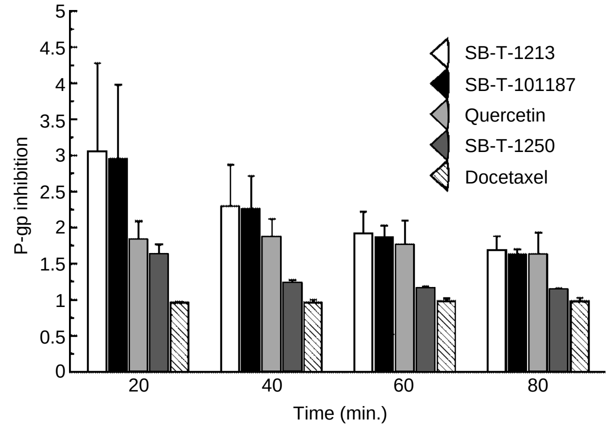

**Figure 5** Bar chart of P-gp inhibition of the panel of taxanes. MCF-7 ADRr cells were loaded with Rh123 (0.5 μM). After 15 min., cells were transferred onto ice and washed twice in cold PBS/BSA 0.2%. On to ice was added 10 μM of all the tested drugs or the vehicle DMSO 0.1%. Cells were then kept at 37˚C to allow the maximal dye efflux.An aliquot of cells was maintained on ice to have the maximal dye uptake. At each time point (from 20 to 80 min), Rh123 fluorescence was assessed by flow cytometry.
The ratio between control at 37˚C and control at 4˚C served to calculate the control dye efflux. The same procedure was employed for each drug. This ratio was divided by the amount of the control dye efflux. A value higher than 1 means that drug acts as P-gp inhibitor. Results obtained from the mean of two independent experiments are shown. Bars represent the standard deviations

### ACKNOWLEDGEMENTS

This work was supported by a grant from the National Institutes of Health, USA (GM42798 to IO).

*© 2000 Cancer Research Campaign* 

### REFERENCES

Bhalla K, Huang Y, Tang C, Self S, Ray S, Mahoney ME, Ponnathpur V, Tourkina E, Ibrado AM, Bullock G et al (1994) Characterization of a human myeloid leukemia cell line highly resistant to taxol. *Leukemia* **8**: 465–475

Chevillard S, Pouillart P, Beldjord C, Asselain B, Beuzeboc P, Magdelenat H and Vielh P (1996) Sequential assessment of multidrug resistance phenotype and measurement of S-phase fraction as predictive markers of breast cancer response to neoadjuvant chemotherapy. *Cancer* **77**: 292–300

Darzynkiewicz Z, Juan G, Li X, Gorczyca W, Murakami T and Traganos F (1997) Cytometry in cell necrobiology: analysis of apoptosis and accidental cell death (necrosis). *Cytometry* **27**: 1–20

De Vincenzo R, Scambia G, Benedetti PP, Fattorossi A, Bonanno G, Ferlini C, Isola G, Pernisco S and Mancuso S (1996) Modulatory effect of tamoxifen and ICI 182,780 on adriamycin resistance in MCF-7 human breast-cancer cells. *Int J Cancer* **68**: 340–348

Distefano M, Scambia G, Ferlini C, Gaggini C, De Vincenzo R,Riva A, Bombardelli E, Ojima I, Fattorossi A, Panici PB et al (1997) Anti-proliferative activity of a new class of taxanes (14beta-hydroxy-10-deacetylbaccatin III derivatives) on multidrug-resistance-positive human cancer cells. *Int J Cancer* **72**: 844–850

Ferlini C, Di Cesare S, Rainaldi G, Malorni W, Samoggia P, Biselli R and Fattorossi A (1996) Flow cytometric analysis of the early phases of apoptosis by cellular and nuclear techniques. *Cytometry* **24**: 106–115

Gao Z, Fields JZ and Boman BM (1999) Tumor-specific expression of anti-mdr 1 ribozyme selectively restores chemosensitivity in multidrug-resistant colon-adenocarcinoma cells. *Int J Cancer* **82**: 346–352

Herrington JD, Di-Nunno L and Rinehart JJ (1998) Lack of CNS penetration of docetaxel in a patient with leptomeningeal carcinomatosis. *Ann Pharmacother* **32**: 611–612

Horwitz SB, Cohen D, Rao S, Ringel I, Shen HJ and Yang CP (1993) Taxol: mechanisms of action and resistance. *J Natl Cancer Inst Monogr Volume*: 55–61

Jordan MA, Wendell K, Gardiner S, Derry WB, Copp H and Wilson L (1996) Mitotic block induced in HeLa cells by low concentrations of paclitaxel (Taxol) results in abnormal mitotic exit and apoptotic cell death. *Cancer Res* **56**: 816–825

Kavallaris M, Kuo DYS, Burkhart CA, Regl DL, Norris MD, Haber M and Horwitz SB (1997) Taxol-resistant epithelial ovarian tumors are associated with altered expression of specific beta-tubulin isotypes. *J Clin Invest* **100**: 1282–1293

Kaye SB (1998) Multidrug resistance: clinical relevance in solid tumours and strategies for circumvention. *Curr Opin Oncol* **10 Suppl 1**: S15–S19

Long BH and Fairchild CR (1994) Paclitaxel inhibits progression of mitotic cells to G1 phase by interference with spindle formation without affecting other microtubule functions during anaphase and telephase. *Cancer Res* **54**: 4355–4361

Manfredi JJ, Parness J and Horwitz SB (1982) Taxol binds tocellular microtubules. *J Cell Biol* **94**: 688–696

Mechetner E, Kyshtoobayeva A, Zonis S, Kim H, Stroup R, Garcia R, Parker RJ and Fruehauf JP (1998) Levels of multidrug resistance (MDR1) P-glycoprotein expression by human breast cancer correlate with in vitro resistance to taxol and doxorubicin. *Clin Cancer Res* **4**: 389–398

Motulsky HJ and Ransnas LA (1987) Fitting curves to data using nonlinear regression: a practical and nonmathematical review. *FASEB J* **1**: 365–374

Naito M and Tsuruo T (1998) Therapeutic approach to drug resistant tumors. *Ther Drug Monit* **20**: 577–580

Ojima I, Slater JC, Michaud E, Kuduk SD, Bounaud PY, Vrignaud P, Bissery MC, Veith JM, Pera P and Bernacki RJ (1996) Syntheses and structure-activity relationships of the second-generation antitumor taxoids: exceptional activity against drug-resistant cancer cells. *J Med Chem* **39**: 3889–3896

Ojima I, Slater JC, Kuduk SD, Takeuchi CS, Gimi RH, Sun CM, Park YH, Pera P, Veith JM and Bernacki RJ (1997a) Syntheses and structure-activity relationships of taxoids derived from 14 beta-hydroxy-10-deacetylbaccatin III. *J Med Chem* **40**:267–278

Ojima I, Kuduk SD, Pera P, Veith JM and Bernacki RJ (1997b) Synthesis and structure-activity relationships of nonaromatic taxoids: effects of alkyl and alkenyl ester groups on cytotoxicity. *J Med Chem* **40**: 279–285

Ojima I, Kuduk SD and Chakravarty S (1998) Recent advances in the medicinal chemistry of taxoid anticancer agents. In: Maryanoff BE, Reitz AB (eds). *Advances in Medicinal Chemistry*, Vol 4. pp 69–124. JAI Press Inc: Greenwich, CT

Ranganathan S, Benetatos CA, Colarusso PJ, Dexter DW and Hudes GR (1998) Altered beta-tubulin isotype expression in paclitaxel-resistant human prostate carcinoma cells. *Br J Cancer* **77**: 562–566

Rao S, Horwitz SB and Ringel I (1992) Direct photoaffinity labeling of tubulin with taxol. *J Natl Cancer Inst* **84**: 785–788

Schiff PB, Fant J and Horwitz SB (1979) Promotion of microtubule assembly in vitro by taxol. *Nature* **277**: 665–667

Shapiro AB and Ling V (1997) Effect of quercetin on Hoechst 33342 transport by purified and reconstituted P-glycoprotein. *Biochem Pharmacol* **53**: 587–596

Smeets M, Raymakers R, Vierwinden G, Pennings A, van-de-Locht L, Wessels H, Boezeman J and de-Witte T (1997) A low but functionally significant MDR1 expression protects primitive haemopoietic progenitor cells from anthracycline toxicity. *Br J Haematol* **96**: 346–355

Torok M, Gutmann H, Fricker G and Drewe J (1999) Sister of P-glycoprotein expression in different tissues. *Biochem Pharmacol* **57**: 833–835

Uchida N, Combs J, Chen S, Zanjani E, Hoffman R and Tsukamoto A (1996) Primitive human hematopoietic cells displaying differential efflux of the rhodamine 123 dye have distinct biological activities. *Blood* **88**: 1297–1305

Verweij J, Clavel M and Chevalier B (1994) Paclitaxel (Taxol) and docetaxel (Taxotere): not simply two of a kind. *Ann Oncol* **5**: 495–505

Yu DS, Chang SY and Ma CP (1998) The expression of mdr-1-related gp-170 and its correlation with anthracycline resistance in renal cell carcinoma cell lines and multidrug-resistant sublines. *Br J Urol* **82**: 544–547

*© 2000 Cancer Research Campaign*
